# CBCT Evaluation of the Root Canal Filling Removal Using D-RaCe, ProTaper Retreatment Kit and Hand Files in curved canals

**Published:** 2014-12-24

**Authors:** Zahra Sadat Madani, Narges Simdar, Ehsan Moudi, Ali Bijani

**Affiliations:** a * Department of Endodontics, Dental School, Babol University of Medical Sciences, Babol, Iran; *; b *Department of Endodontics, Dental School, Guilan University of Medical Sciences Rasht, Iran; *; c *Department of Oral and Maxillofacial Radiology, Dental School, Babol University of Medical Sciences, Babol, Iran; *; d * Non-Communicable Pediatric Diseases Research Center, Babol University of Medical Sciences, Babol, Iran*

**Keywords:** CBCT, Cone-Beam Computed Tomography, Endodontic Retreatment, Obturation materials, Root Filling Materials

## Introduction

**Introduction:** The aim of the present *in vitro* study was to evaluate the efficacy of D-RaCe, ProTaper retreatment kit and hand H-files in removal of obturating materials (OM) from the curved root canals using cone-beam computed tomography (CBCT). **Materials and methods:** Sixty extracted molars were prepared and obturated. The samples were divided into three groups (*n*=20). In each group the OM was removed using hand H-files, D-RaCe and ProTaper retreatment kit. All the samples underwent CBCT imaging. The amount of OM was evaluated in CBCT sagittal cross-sections and scored. The maximum concentration of residual OM was recorded. The duration of the procedure (including the required time for reaching working length=T1 and total working time=TT) and procedural errors were also recorded. Data were analyzed using one-way ANOVA, Tukey’s post hoc test, Fisher’s exact test and Kruskal-Wallis test. The level f significance was set at 0.05. **Results:** No significant differences were observed in the residual OM among the three groups. T1 and TT were not significantly different in all groups. There were no significant differences in concentration of OM between the groups (P<0.05). In relation to procedural errors, 4 and 5 cases of file fracture were recorded in the ProTaper and D-RaCe groups, respectively, with no significant differences. **Conclusion:** Rotary and hand H-files had similar efficacy in removing root canal filling materials but instrument fracture occurred more frequently in rotary files.

## Introduction

Most of the complications of endodontic treatment are attributed to the persistence of bacteria within the root canal system mostly due to inadequate debridement, undetected and untreated root canals, inadequate obturation or coronal leakage [1]. Gutta-percha is the most commonly used root canal obturation material (OM), which offers the advantage of easy removal during endodontic retreatment. Removal of the root OM from the curved canals is much harder. It is usually extirpated by hand H-files alone or in combination with Gates-Glidden drills, with or without a solvent [2]. Other OM removal techniques include the use of hot instruments, engine driven files, ultrasonic instruments and laser [3, 4]. 

H-files are formed by cutting a continuous flute in a tapered wire. H-files files cut the canal walls when they are pulled out or rotated in a clockwise direction [5]. A large number of studies have shown that after endodontic retreatment, a considerable volume of root canal OM remain in the root canal which must be removed because the possibly of compromising the seal and harboring bacteria [6-9]. 

In many studies it is stated that the use of nickel-titanium (NiTi) rotary instruments is a safe and efficient way of removing root canal OM during endodontic retreatment [4, 8, 10, 11]. Rotary instruments require less time to clean the canals compared to hand instruments; therefore, both the patient and operator would benefit from less chair time [10, 12, 13]. The safety and cleaning efficacy of rotary instruments is also proved [14-16]. 

ProTaper Universal retreatment kit (Dentsply Maillefer, Ballaigues, Switzerland) include D1 (30/0.09), D2 (25/0.08) and D3 (20/0.07) files with different tapers and tip diameters which are specifically designed to remove the root canal OM from the coronal, middle and apical thirds of the canals, respectively [13]. Similar to finishing and shaping ProTaper instruments, retreatment files have a convex cross-section [8]. 

The D-RaCe retreatment system (FKG Dentaire, La-Chaux-de Fonds, Switzerland) consists of two instruments: DR1 (30/0.10) with an active tip to facilitate penetration into the OM for clearing the coronal segment of the canal and DR2 (25/0.04) that reaches the working length (WL) [17].

Use of cone-beam computed tomography (CBCT) in endodontics has been common in recent years and it has exhibited better efficacy compared to routine radiographic techniques in the diagnosis of apical periodontitis, evaluation of the root canal system, resorptive lesions and in treatment planning of endodontic surgery [4, 18]. 

The present *in vitro* study was performed to evaluate and compare the efficacy of H-files, D-RaCe and ProTaper retreatment kit in removing gutta-percha from the curved root canals, by means of CBCT.

## Materials and Methods

Mesial and mesiobuccal roots of 60 extracted mandibular and maxillary molars were selected for this study. Teeth with cracks, resorption, immature apices and S-shaped curvatures were excluded from the study. The curvature of the canal and the radius of the curvature were calculated using the method offered by Schafer and Schneider and the means were determine [19, 20]. Only teeth with a minimum canal curvature of 20° and a maximum radius of curvature of 12 mm were selected (Table 1). Soft tissue and calculi were mechanically removed from the root surfaces and the teeth were disinfected with 2% gultaraldehyde solution. After preparation of access cavity, a #10 stainless steel K-file (Dentsply Maillefer, Ballaigues, Switzerland) was placed in the root canal so that the file tip was visible at the apical foramen. Digital radiographic images were prepared by PSP sensors (Soredex; Orion Corporation Ltd., Helsinki, Finland) in the buccolingual and mesiodistal directions and processed by Digora PCT scanner (Soredex; Helsinki, Finland) software.

In order to standardize all the teeth, the teeth were decoronated to reach a root length of 18 mm and a WL of 17 mm was chosen for all the teeth. All the samples were prepared by the same operator using ProTaper instruments up to F2 file installed in an electric motor (Endo-Mate TC, NSK, Nakanishi Inc., Tokyo, Japan) with a speed of 300 rpm and 3 N/m torque. The canals were irrigated with 1% NaOCl carried into the canal with a 30-guage needle between files. After completion of preparation, the smear layer was removed with 5 mL of 17% EDTA and 5 mL of 1% NaOCl as the final rinses and dried with paper points. The root canals were obturated with gutta-percha cones covered with AH-Plus sealer (Dentsply, De Trey, Konstanz, Germany) using the cold lateral compaction technique; until the last lateral cone could not penetrate into the canals more than 5 mm. A hot plugger was used to remove extra gutta-percha. The quality and the apical extension of the obturation were evaluated by radiography in the buccolingual and mesiodistal directions. The post obturation images were processed by Digora PCT software on a computer. 

At this stage, all the primary CBCT images were taken using NewTom VGi unit (QR SRL Co., Verona, Italy). The access cavity was temporarily sealed. Then the samples were stored at 37^°^C and 100% relative humidity for 14 days for the complete setting of the sealer. Then the samples were randomly allocated into groups 1 to 3 (*n*=20) according to the retreatment technique. In all groups the temporary restoration was removed and one drop of chloroform was used for softening the first 2 mm of the OM. 

***Group 1 (H-file):*** The coronal third of the OM was removed with sizes 3 and 2 of Gates-Glidden drills (Mani, Tochigi, Japan) at 2000 rpm. Then the rest of the OM was removed with #30, 25 and 20 of H-files (Dentsply Maillefer, Ballaigues, Switzerland) in a descending order and with circumferential filing and quarter-turn push-pull movements until they reached the WL. 

***Group 2 (D-RaCe):*** D-RaCe files were used with a speed/torque of 600 rpm and 1 N/m, respectively. The coronal third of the OM was removed with DR1 file (30/0.10). Then the DR2 file (25/0.4) was used in an apical direction to the WL. Based on manufacturer’s instructions, the latest file was single used. Then the apical preparation was done with BR3 (25/0.06) and then BR4 (35/0.04) instrument (BioRaCe, FKG Dentaire, La Chaux-de-Fonds, Switzerland). 

***Group 3 (ProTaper retreatment kit):*** ProTaper files were used at a speed of 300 rpm with a 3 N/m torque. The coronal third of the canal was cleaned with ProTaper D1 file (30/0.09). The OM in the middle and apical thirds was removed with D2 (25/0.08) and D3 (20/0.07) files, respectively. Preparation of the apical area was carried out with F2 (25/0.08) and F3 (30/0.09) instruments.

**Table 1:** The mean (SD) of root canal curvatures in all three study groups (*n*=20)

**Group**	**Canal curvature** **in degrees**		**Canal radius in mm**	
	**BL**	**MD**	**BL**	**MD**
**H-file**	22.95 (4.23)	22.49 (2.70)	9.86 (1.56)	10.13 (1.73)
**D-RaCe**	22.41 (2.15)	22.49 (1.47)	10.38 (1.30)	9.80 (1.42)
**ProTaper**	23.56 (4.36)	22.43 (1.60)	9.84 (1.69)	10.27 (1.07)
***P*** **-value**	0.598	0.995	0.500	0.561

**Figure 1:**
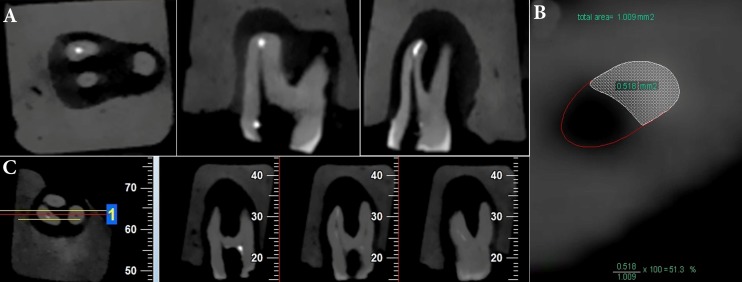
*A)* Axial, coronal and sagittal CBCT cross-section; *B)* calculation of the surface areas; *C)* The cross-sections used for observational scoring

Probable procedural errors, including perforation, ledges, blockades and instrument fracture were recorded. In case of instrument fracture, a hand file was used to remove the separated segment. The time spent to remove the broken instrument was not included in the working time. The final rinse was carried out by 5 mL of 20% EDTA and 5 mL of 1% NaOCl. Then the root canals were dried with paper points. 

A chronometer was used to determine the time needed to reach the working length (T1) and the time needed to completely remove the OM and make a final preparation (T2). The time needed to change instruments and irrigate the canals was not calculated and recorded. The overall time recorded (TT) was recorder by the sum of T1 and T2. Complete removal of OM was confirmed when no gutta-percha or sealer was observed on the flutes of the instrument or during irrigation.

Then post-retreatment CBCT images were taken and evaluated in axial, coronal and sagittal plans (Figure 1A). CBCT cross-sections were 1 mm thick and were taken from 0.5 mm segments of the canal from apical to coronal at 0.5, 1, 1.5, 2, 2.5, 3, 3.5 and 4-mm levels within the canal using the NTT Viewer program (NTT Software Corporation, Yokohama, Japan). The root length of each sample was divided into 3 areas of apical, middle and coronal in these cross-sections and the percentage of residual obturation material on the walls in each area was calculated at 1-mm distances from the apical area toward the canal orifice using the AutoCAD software (Autodesk Inc., San Rafael, CA), using the following formula: (S1/S2)×100, where S1 is the surface area of the residual OM and S2 is the surface area of the root canal [1] (Figure 1B).

In addition, the highest concentration of residual gutta-percha in the three apical, middle and coronal areas of the CBCT cross-sections and in different areas of the root canal walls (buccal, lingual, mesial and distal) was recorded.

Furthermore, sagittal CBCT cross-sections (Figure 1C) were used for observational scoring of the residual obturation material based on the scoring system introduced by Somma *et al.* [21] as follows: *score 1*-no residual material or a small amount of residual material on the dentin surface (<25%); *score 2*-some residual debris on the dentin surface (25‒50%); *score 3-*a moderate amount of debris on the dentin surface (50‒75%); *score 4*-a large amount of debris on the dentin surface (>75%).

During scoring, the CBCT cross-sections were first evaluated by an observer and then confirmed by the second observer. During all the evaluations carried out no attempts were made to make a distinction between the obturation material and sealer. The data was analyzed using the SPSS software (SPSS version 17, SPSS, Chicago, IL, USA). The one-way ANOVA test was used to determine the mean of quantitative data. Tukey’s post hoc test was used to evaluate the difference between the groups. Fisher’s exact test was used to determine the differences between groups. Moreover the Fisher’s exact test and Kruskal-Wallis test were used to compare the nominal data and evaluation of data with normal distribution, respectively. The level of significance was set at 0.05.

## Results

The study was carried out on 60 extracted mandibular and maxillary molars. The mean root canal curvatures in the buccolingual and mesiodistal directions were 22.97±3.69 and 22.47±7.97 degrees, respectively; the mean radii of curvature in the buccolingual and mesiodistal directions were 10.03±1.52 and 10.06±1.42 mm, respectively. Table 1 presents the amounts of root canal curvatures in the three study groups. The ANOVA analysis revealed no significant differences between the groups in canal curvatures.

**Figure 2:**
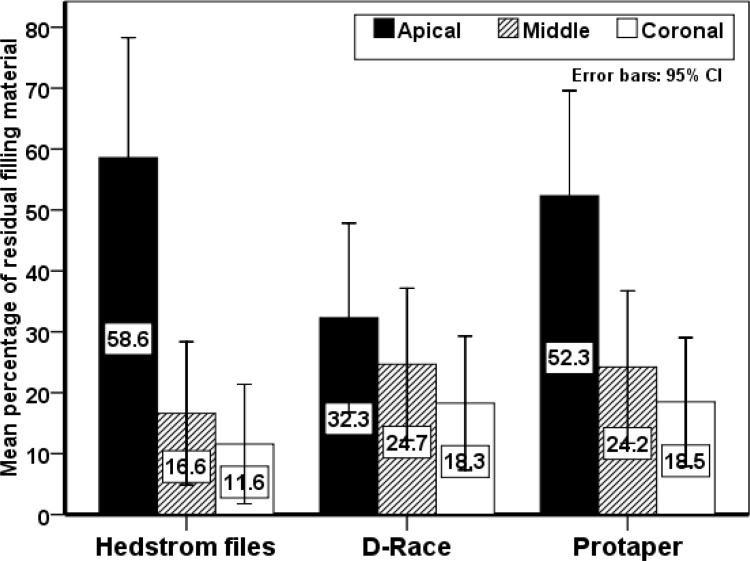
The percentages of the residual obturation materials remaining in each retreatment group

There were no significant differences in residual OM in the apical, middle and coronal areas between the three retreatment techniques (*P*<0.05); however, in the H-file group, the residual OM in the apical third was significantly more than that in the middle and coronal thirds (*P*=0.035). In addition, in the ProTaper group, the residual OM in the apical area was significantly more than that in the middle and coronal thirds; however, the differences were not significant in the D-RaCe group. Evaluations showed that none of retreatment techniques was able to completely eliminate the root canal filling material from the root canal (Figure 1A). Figure 2 presents the percentages of the OM remaining in the apical, middle and coronal thirds.

Table 2 presents the amounts of T1, T2 and TT in study groups. There were no significant differences between the three retreatment techniques. However, there was a significant difference in the length of T2 in the H-file group which had shorter T2 time compared to other groups.

In addition, the highest concentrations of residual OM in different areas of the root canal walls (buccal, lingual, mesial and distal) were evaluated on CBCT axial cross-sections, with no significant differences between the three study groups. 

**Table 2:** Means (SD) of T1, T2 and TT in seconds (T1=time needed to reach the working length, T2=the time needed to completely remove the OM and reach the final preparation, TT=T1+T2)

	**H-file**	**D-RaCe**	**ProTaper**	***P*** **-value**
**T1**	271.75 (75.57)^a^	247.90 (88.86)^a^	257.20 (66.19)^a^	0.620
**T2**	138.75 (69.43)^a^	212.10 (81.06)^b^	201.40 (81.007)^b^	0.008
**TT**	410.5 (96.20)^a^	460.0 (98.64)^a^	458.6 (99.23)^a^	0.201

* *Similar letters indicate insignificant differences*

Table 3 presents the observational scores on CBCT cross-sections in relation to the amount of residual OM in the entire canal length on sagittal cross-sections, with no significant differences between the three groups. In addition, there was no significant difference in the observational scores between the two observers.

No procedural errors were observed with H-files. However, 4 and 5 cases of file fractures were observed with ProTaper and D-RaCe files and the in this regard both rotary groups were similar.

## Discussion

This study showed that there were no significant differences in the amount of residual OM among H-file, D-RaCe and ProTaper retreatment instruments. Moreover, it was shown that H-files required less time compared to rotary files for cleaning the root canal system.

The success of non-surgical endodontic retreatment depends on the elimination of necrotic tissues, bacteria and contaminated previous OM from the root canal system [22]. Therefore, extirpation of the previous OM is necessary in gaining access and decontaminate all areas of root canal system [23]. 

Similar studies on the amount of residual OM within the root canals were mostly carried out on straight roots [8, 10, 14, 24, 25]. However, many other studies have used curved canals [7, 11, 26, 27]. In the present study, the root canal curvatures and radii of curvature were similar in all the three study groups. In order to standardize the samples the samples were decoronated. Also the root canals were shaped in a similar manner in all the samples. 

The root canals were obturated with gutta-percha and sealer using lateral compaction technique similar to other studies [7, 8, 25]. AH-Plus sealer was used in the present study, which can bind to canal dentin [28]. The same as other similar studies, none of the retreatment techniques in the present study were able to completely remove the OM from the root canal [2, 4, 17]. There were no significant differences in the percentages of residual OM among the H-file, D-RaCe and ProTaper samples. In a study by Dall’agnol *et al.* [28], no significant differences were observed between manual instruments, ProTaper retreatment files and Reciproc and the large amount of residual gutta-percha was attributed to the binding of AH-Plus sealer to the root dentin and the complex anatomy of the root canal system, making it difficult to remove the obturation materials. In a study by Rodig *et al.* [29], the efficacy of hand and rotary files in removing gutta-percha from curved root canals were compared using micro-computed tomography (µ-CT). Their results showed that hand files left significantly less OM during retreatment, which is different from the results of the present study; the difference might be attributed to differences in the internal anatomy of the samples. 

**Table 3:** Percentage scores of the residual obturation material (percent) by observational analysis (*P*=0.082)

	**H-file**	**D-RaCe**	**ProTaper**
**Score 1**	90.0	70.0	60.0
**Score 2**	10.0	15.0	25.0
**Score 3**	0	15.0	15.0
**Score 4**	0	0	0

The results of the present study showed that retreatment with H-files required less time compared to rotary files, which might be attributed to a higher efficacy of H-files in removing gutta-percha in one bulk after its entanglement in the file flutes. It has already been reported that rotary files require less time during retreatment compared to hand files [2] and Bramante *et al.* [30], attributed this shorter time to the plasticity of gutta-percha with the use of rotary instruments and therefore, the softer removal of the OM. However, this condition of gutta-percha with the use of rotary files might lead to the adhesion of gutta-percha to canal walls, especially in curved areas, making it difficult to remove OM. In a study by Rodig *et al.* [31], rotary and reciprocating files in curved canals required less time compared to hand instruments. 

In a study by Hulsmann *et al.* [23], use of pre-curved hand files facilitated the removal of gutta-percha by improving tactile sensation, which was recommended as an adjunct to rotary files during retreatment. The results of the present study showed that none of the retreatment techniques can guarantee complete removal of gutta-percha, which is consistent with previous studies [17]. However, Çelick *et al.* [26] reported lower efficacy of ProTaper files compared to hand files in curved canals and attributed this superiority to canal enlargement beyond the D3 (20/0.07) retreatment file based on manufacturer’s instructions; however, #30 hand K-file exhibited higher efficacy in removal of the OM from the root canal compared to rotary files with greater size and taper. 

In the present study, some procedural errors and file fractures were observed in the rotary file groups, which might be attributed to the great taper of rotary files and low radius of curvature of root canals, which are regarded as definite factors in possible fracture of rotary instruments[17]. 

Other studies, have also shown the high risk of rotary instrument fracture compared to hand files [11]. In the present study, no errors were observed in the H-files which might be attributed to the tactile sensation of hand files. The fractured rotary files were removed with hand files, which resulted in more debridement in the affected root canals and changes in the standard deviation. An attempt was made to evaluate the highest concentration of residual OM in different areas of the canal walls (buccal, lingual, mesial and distal) in the CBCT axial cross-sections, which did not reveal any significant differences, indicating that the residual OM might remain in every area of the root canal. The amount of residual OM depends on the operator, root canal anatomy and type and quality of previous root canal obturation.

In general, the results of the present study regarding the amount of residual OM during retreatment with hand files and D-RaCe and ProTaper rotary instruments did not exhibit significant differences and retreatment with rotary files resulted in a higher rate of procedural errors. 

## Conclusion

None of the retreatment techniques could completely remove the obturating material from the root canal. Moreover, there was no superiority regarding the efficacy of root filling removal. Since there is a high rate of procedural errors with the use of rotary files, their using with adjunctive hand instrumentation might be useful. 
